# PM_2.5_ and Asthma Disparity in Relation to Social Vulnerability Index: A Case Study from Durham, North Carolina

**DOI:** 10.3390/toxics14030221

**Published:** 2026-03-04

**Authors:** Macie D. Bethea, Sterling Brown, Sara Harrison, John Bang, James Harrington, Vijay Sivaraman

**Affiliations:** 1 Biological and Biomedical Department, North Carolina Central University, Durham, NC 27707, USA; mbethe16@eagles.nccu.edu (M.D.B.); sbrow217@eagles.nccu.edu (S.B.); jjbang@nccu.edu (J.B.); 2 Center for Analytical Sciences, RTI International, Durham, NC 27709, USA; seharrison@rti.org (S.H.); jharrington@rti.org (J.H.)

**Keywords:** PM2.5, TEM, EDX, asthma, disparities

## Abstract

Increased air pollution and associated disease prognosis are a serious concern in communities across the socioeconomic spectrum. Past studies have shown that a major component of air pollution, fine particulate matter (PM_2.5_), is elevated in majority-Black communities in the US to greater levels than those in majority-White communities, which can potentially contribute to higher rates of respiratory health issues. In this study, we address whether PM_2.5_ correlates with increased asthma rates in Durham, North Carolina. We selected monitoring sites in different census tracts within the same zip code with disparate levels of asthma to quantify and characterize PM_2.5_ levels. We found that South Durham, which has higher asthma hospitalization rates, has higher average PM_2.5_ levels (78.49 µg/m^3^) than North Durham (26.3 µg/m^3^). We measured the elemental composition of PM samples using transmission electron microscopy–energy dispersive X-ray spectroscopy (TEM-EDX) and found significant differences in the levels of Na, S, Ca, Mg, Fe, and Ti. Our data suggests that these differences in ambient PM_2.5_ could contribute to differences in health outcomes in the two areas. We also discuss these differences in the context of social vulnerability within the two study sites and show that the more vulnerable site (South Durham) experiences higher pollution rates.

## 1. Introduction

Pulmonary illness is among the leading causes of death globally, driven by several contributors, including tobacco use, allergens, occupational exposures, and air pollution (CDC MMWR). One subclass of air pollutant that has been shown to contribute significantly to the risk of pulmonary illness is the fraction called particulate matter (PM). PM is a broad term that refers to free-floating microscopic particles in the air. These particles can be natural (e.g., dust, dirt, or pollen) or anthropogenic (such as vehicle emissions or carbon-based power plant emissions) and contain a range of elemental compositions, including sulfates and organic carbon, depending on the specific source. One particularly hazardous type of PM is fine particulate matter, also called PM_2.5_ because the particles are 2.5 microns in size or smaller in aerodynamic diameter. This complex portion consists of both solid particles and liquid droplets that can penetrate deeply into the lungs when inhaled, producing inflammation and respiratory complications. PM_2.5_ is commonly produced by the burning of wood, coal, and heating oil and is also considered to be one of the most detrimental forms of PM by the US Environmental Protection Agency [1,2]. Its chemical composition can vary significantly depending on the source of emission and the origin of the fuel, but sulfate, nitrate, ammonium, silicon, and sodium ions and organic and elemental forms of carbon (OC and EC, respectively) are some commonly observed chemical components that can impact environmental and human health [3]. PM can also contain toxic trace elements that have been linked to cancer and neurological issues like manganese, chromium, lead, arsenic, and cadmium, although these components only make up about 1% each of a total PM_2.5_ sample.

Levels of PM_2.5_ in the air can vary significantly due to several factors, including location (e.g., proximity to sources, prevailing weather patterns, and availability of vegetation) and seasonality [4,5]. Most notably, PM_2.5_ tends to be most prevalent in urban areas as opposed to more rural areas in the United States [6]. An extensive body of work has shown inverse correlations between income and other socioeconomic indicators and exposure to pollutants, including those in ambient air [7,8,9]. Studies have also shown seasonal variability in PM_2.5_ concentrations [6,10]. On a single day, PM_2.5_ can be influenced by factors including average traffic volume and precipitation. Some factors that can contribute to these differences are colder air temperature in the winter and less wind in the fall and winter compared to warmer seasons.

Differences in exposure patterns to pollutants in the air or other matrices have been shown to contribute to differential health outcomes, commonly referred to as health disparities [11]. Health disparities remain a critical concern in public health, as they are recognized to reflect complex systemic phenomena that reflect the interconnected nature of environmental exposures, health care accessibility, community support systems, and historical power structures. Vulnerable populations, particularly those with lower incomes and racial or ethnic minorities, are often disproportionately exposed to environmental hazards due to their proximity to industrial zones, major highways, and substandard housing infrastructure [12]. Minoritized groups, especially Black and Hispanic populations in the US, are more likely to reside in areas with elevated pollution levels [13]. Such environmental exposures exacerbate existing health disparities, particularly in respiratory health [14,15]. Pollution emitters are more frequently located within areas where communities of color reside (such as industrial areas and highways), leading to greater exposure to airborne contaminants like particulate matter and ozone [11]. This disproportionate exposure is associated with higher rates of asthma and other respiratory diseases. Black Americans, in particular, experience increased rates of asthma-related hospitalizations and mortality compared to their White counterparts [13]. Health disparities can also be attributed in part to social determinants of health, including poverty and residential segregation resulting from the historical practice of redlining [16]. Socioeconomic status (SES) also plays a significant role in determining respiratory health outcomes. Individuals from lower-income backgrounds face a confluence of risk factors, including limited access to healthcare, green spaces, and healthy food; higher environmental exposures; and social/emotional trauma that can impact health and wellbeing. Understanding the cumulative impacts of these environmental and socioeconomic factors, both in the present and over time, can provide a more detailed assessment of their effects on the health of the community [17]. People in low-SES communities are more likely to be exposed to harmful air pollutants [7], which can aggravate conditions like asthma and chronic obstructive pulmonary disease (COPD). Children from economically disadvantaged households are particularly vulnerable, often experiencing higher exposure to indoor allergens and secondhand smoke—factors that directly contribute to increased asthma incidence. Those with lower incomes are less likely to have adequate health insurance, reducing their access to preventive care and timely treatment for respiratory issues. This lack of access can result in more frequent emergency room visits and hospitalizations, compounding the overall health burden. While some of the reasons for health inequities may have historical and societal underpinnings, becoming aware of the causative nature of exposures to disease is a first step towards identifying interventions.

We previously analyzed inpatient and emergency department discharge data in North Carolina over the twelve 5-digit billing zip codes of Durham, NC, from 2012 to 2017 and found that zip code 27701 carried the highest burdens of asthma incidence/exacerbations [18]. Overlaying this data with US Census data provided visualization of distinct distributions of Black American residence, which ranged between 15 and 89%. Two census tracts carrying highly differential populations were selected within the zip code for further assessment of PM_2.5_ [19]. We quantified and characterized the composition of particulate matter from these sites to explore the hypothesis that inhaled environmental pollutants such as PM_2.5_ contribute to the observed disparities in rates of pulmonary illness between these two areas of Durham.

Community partnerships were developed to identify outdoor air collection at seasonal periods. From these sites, we aimed to quantify and collect particulate matter. The collected particulate matter was also analyzed by transmission electron microscope to characterize its composition. By collecting and analyzing PM_2.5_ from various locations, we aim to develop a better understanding of its potential impact on outdoor air quality and ultimately support our assessment of environmental contributors to respiratory health disparities in Durham.

## 2. Methods

### 2.1. Purple Air and PEM Unit Organization

**Figure 1:**
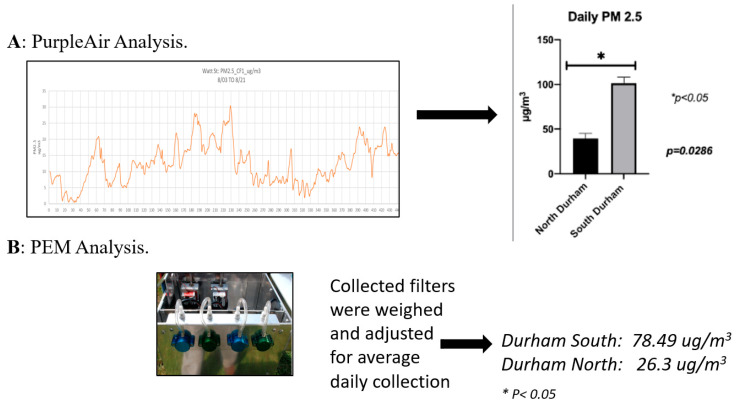
Particulate matter (PM_2.5_) quantitation. PM_2.5_ was quantitated by PurpleAir systems and PEM collection systems (*N* = 3). * *p* < 0.05.

Sample sites were determined as described in Richey et al., 2024 [18]. To measure real-time PM_2.5_ concentrations in diverse neighborhoods, PurpleAir monitors (PA-II, Draper, UT, USA) were installed at two different sites within Durham. These monitors operated concurrently with the PEM units, enabling continuous tracking of ambient PM_2.5_ levels. For this study, we used personal environmental monitors to collect PM for analysis of concentration and composition. Personal Environmental Monitoring (PEM; SKC (Eighty Four, PA, USA) Airchek Model XR5000) units were used to collect air samples, and PM_2.5_ was subsequently extracted from these filters for weighing (Figure 1, * *p* < 0.05) and follow-up cellular exposure studies (in preparation). Both air monitors were placed in a fixed outdoor environment roughly 10 m away from the roadway and 1.5–2 m above ground level (the range of typical breathing height). TEFLON filters (Air Quality Engineering Inc., Brooklyn Park, MN, USA) were used in the PEM, and air was sampled at a constant flow rate of 5–10 mL/min. Samples were collected for 2-week intervals, with the units situated roughly adjacent to each other, close to roadways, at the average height of an individual to emulate human inhalation. Additionally, the units were placed under waterproof coverings, and the batteries for each unit were replaced every 24–48 h to ensure consistent sampling quality. We collected particulate samples in two seasons to account for variability in PM concentrations and content.

### 2.2. PM_2.5_ Isolation

By collecting and analyzing PM_2.5_ from various locations, we aimed to develop a better understanding of its potential impact on outdoor air quality and ultimately support our assessment of environmental contributors to respiratory health disparities in Durham. The samples were submitted for analysis in August and November 2023. The PEM filters were gravimetrically analyzed after collection to determine the mass of PM collected. PM was extracted from the filters by submerging them in a 95% ethanol solution and sonicating at 40 kHz for two minutes. The extract solution was transferred to 50 mL centrifuge tubes and centrifuged at 8000× *g* for 15 min. The samples were frozen in liquid nitrogen and concentrated through lyophilization in a FreeZone freeze-dryer (Terra Universal, Fullerton, CA, USA).

### 2.3. TEM/EDX Analysis

The dry PM_2.5_ extracts were resuspended in serum-free media prior to physicochemical characterization by transmission electron microscopy (TEM) and electron dispersion X-ray spectroscopy (EDX), respectively. In total, ~44 particles were analyzed by EDX. The collected samples were deposited on copper grids and stored on separately labeled PetriSlides. Each sample was analyzed on a Hitachi (Hitachi, Tokyo, Japan) HT7820 dual-mode high-resolution, high-contrast transmission electron microscope (TEM), equipped with an AMT NanoSprint 1200 high-definition digital camera for imaging and an Oxford Instruments (Oxford Intruments, Abingdon, UK) Ultim Max EDS detector, operated by AztecTEM version 6.0 for semi-quantitative elemental characterization. The TEM was operated at an 80 kV accelerating voltage in high-contrast mode. Multiple images were obtained from 8000× to 300,000× magnification. Elemental spectra were identified from samples obtained from two collection sites, and roughly 30 particles of each sample were used to calculate the approximate relative weight percent of each element in the sample (* *p* < 0.05). Copper is present in all samples due to the TEM grid, and so it was not reported.

### 2.4. Statistical Analysis

Statistical analyses were performed using GraphPad (Boston, MA, USA) Prism 10 Academic. Unpaired *t*-tests and ANOVA were conducted on each bar graph containing average PM_2.5_ levels from each regional location to determine if there was any statistically significant difference between the compared sample site locations and groups.

## 3. Results

A representative plot of air quality measured in CT 14 is shown in Figure 1A. The collected data demonstrate that South Durham consistently experienced higher ambient PM_2.5_ levels (100 µg/m^3^ average) compared to North Durham (45 µg/m^3^ average) throughout the monitoring period (*p* = 0.0286, Figure 1). Additionally, the time-averaged daily exposure in CT 14 surpassed the USEPA’s 24 h exposure threshold for PM_2.5_ (35 µg/m^3^) on several days during the collection period of several weeks. The averages for both seasons were similar in difference between sites, though more environmental variability was observed during the seasonal shift. These data conformed with gravimetric analysis of the PM collected from the PEM filters, with a significant difference measured between the filters (Figure 1B). These data suggest that residents in South Durham likely face greater health risks over time due to PM_2.5_ exposure, potentially contributing to the region’s higher rates of respiratory illness and resulting in impacts on life expectancy (Table 1). The US Center for Disease Control and Prevention calculated the life expectancy of CT 3.02 as 80.8 years, compared to 70.7 years in CT 3.02 [20].

**Figure 2:**
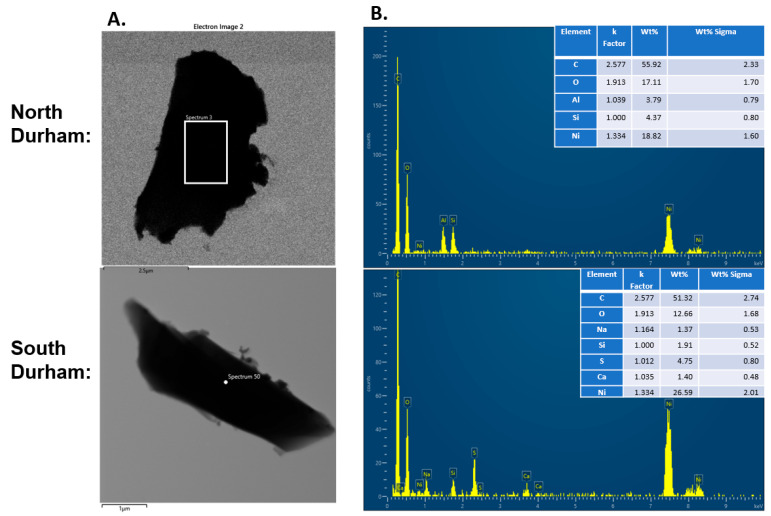
Transmission electron microscopy (TEM) and electron dispersion X-ray spectroscopy. PM collected with a nylon mesh filter (PEM) was isolated and evaluated by TEM analysis. (**A**) Representative image of collected PM_2.5_. (**B**) Representative EDX plot of elemental detection within the sample.

**Figure 3:**
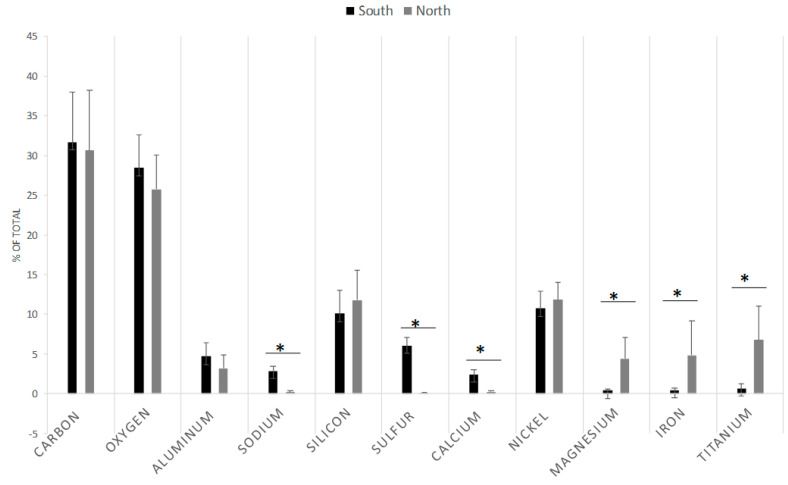
EDX comparison from site collections. Collected data from ~44 images from the North and South Durham collection sites were aggregated and quantified, with significant differences presented between the sites (* *p* < 0.05).

TEM analysis revealed that the collected particulate matter displayed several morphologies, predominantly spherical and irregularly shaped particles with amorphous structures (Figure 2). A representative TEM image captured from particles collected in both North and South Durham is shown in Figure 2A, and one of the EDX spectra measured on the particle with elemental labeling is shown in Figure 2B. Combined EDX analysis of aggregate samples collected from both North and South Durham contained similar major elements, although their relative abundances differed (Figure 3). A comparison of the common elemental profiles from both locations is shown in Figure 3. The North Durham samples had higher concentrations of magnesium, nickel, iron, and titanium. In contrast, the South Durham samples contained greater amounts of sulfur, calcium, and sodium.

## 4. Discussion

Briefly, two census tracts were identified within zip code 27701 using ICD code designations demonstrating high levels of asthma exacerbations and prolonged hospitalization due to pulmonary inflammation. CT 3.02 (North) and CT 14 (South) were chosen for comparison due to the variation in populations that reside in these tracts (Table 1). In addition to the health indicators of the two census tracts, the CDC calculated social vulnerability indexes (SVIs), which are abstract statistics that consider several socioeconomic indicators (e.g., community poverty, transportation availability, and housing conditions) to determine the potential impact on community wellbeing from natural or manmade disasters. There are observable differences between the SVI of the two tracts, with CT 3.02 having an SVI of 0.4717 and CT 14 of 0.9687. This comparison indicates higher vulnerability in CT 14 (the state of NC has an SVI of 0.50) [21].

Drawing on census population data for both tracts allows for the calculation of a disproportionality ratio (DR), which is defined as the ratio of the percentage of a racial/ethnic group in one area to the percentage of the same racial/ethnic group in another area [22]. In this case, the DR is calculated as the percentage of the population that is Black in a census tract compared to the percentage of the population that is Black in the state of NC. The closer the DR is to 1, the more representative a subgroup is compared to the larger area. The DRs of both census tracts compared to the rest of the state are shown in Table 1, demonstrating significant disproportionalities between the two census tracts.

Though a general EPA air quality assessment for Durham County exists [23], official measurements are limited to generalized locations covering large regions. This results in a lack of localized data for communities to critically assess air pollutant levels and health risks for conditions like asthma. Our study demonstrates that a localized approach can be used to gain a more granular understanding of airborne particulate matter at a community level, revealing significant differences between geographically close communities. These differential exposures can also be linked to assessments of sociodemographic factors and assessments of community vulnerability to demonstrate the root causes of health disparities, in this case the link between elevated levels of asthma in an area and higher PM_2.5_ levels in the community.

We measured PM_2.5_ levels in two regions of zip code 27701 in Durham, North Carolina (denoted as North and South Durham) and collected ambient air samples to characterize the chemical composition of the particles. We deployed both PurpleAir monitors and PEM systems in both study locations for a two-week period and monitored real-time concentrations of airborne particulate matter. The results highlight disproportionate exposure to PM_2.5_ between North and South Durham, with South Durham exhibiting significantly higher levels of particulate matter (as measured by both systems). These elevated PM_2.5_ concentrations can be correlated with observed differences in inflammatory responses (i.e., asthma), suggesting a possible link between environmental exposure and observed health outcomes in two communities with disparate racial/ethnic makeups—one of which can be classified as a vulnerable population by the CDC [24,25].

Beyond the concentration of PM, the composition of particles can be related to increased potential for adverse health outcomes [26,27]. A review of clinical literature found studies indicating that sulfate, sodium, and potassium concentrations are significantly positively correlated with mortality and respiratory hospital admission, though it is currently unclear what contribution the elemental crystalline shapes may have on pathology. It is also reported that sulfate and some trace elements in PM can be correlated with cardiovascular and pulmonary macrophage-trophic outcomes [28,29]. Our results indicated that both sulfur and sodium levels were increased in South Durham samples, while titanium, iron and magnesium were higher in North Durham. These levels may be associated with nearby industries as well as traffic from nearby roadways. Prior studies of the elemental composition of roadside PM suggest that Si (similar between both sites) has been noted as a potential tracer for resuspended road dust [30,31]. Ti (elevated in the North Durham site) and Ca (elevated in the South Durham site) may indicate some contribution from either tire wear or from resuspended road dust. Ni (also comparable in both sites) may indicate a contribution from brake wear particles. However, it should be noted that the elements reported here are primarily major constituents and that a more detailed characterization of the trace elemental concentration and speciation analysis, with more samples collected over time (and wind pattern analysis), will allow for a more detailed analysis of the potential health risks caused by exposure to particulate matter.

The findings reported here are consistent with previous research indicating that minoritized communities often face a disproportionate burden of environmental pollution due to many factors, including proximity to industrial areas and highways. The presence of a major highway (147) as well as the urban planning approach in South Durham likely contributes to this phenomenon. Furthermore, the results underscore the need for continued investigation into the chemical composition of PM_2.5_ in different regions and its long-term impact on respiratory and overall public health.

While our study provides important environmental and chemical information to support human health risk assessment, several factors limit the breadth of our findings, which can be addressed in future work. Weather variability always poses a challenge, particularly precipitation, as it can exacerbate short-term variability in PM levels and potentially damage instrumentation. To mitigate this concern in the current study, all particulate monitoring was conducted on days with no rainfall, and several sample collection days were halted early due to unexpected rainstorms. Monitoring over longer time periods can mitigate the risk of influencing the results in future studies.

## 5. Conclusions

Community-engaged environmental health research involving marginalized groups can potentially encounter resistance and low participation due to lack of trust and relationship building. Historical and systemic injustices have contributed to deep-rooted mistrust in medical and scientific research among some low-income and minority communities [32]. This hesitancy makes it more difficult to foster collaboration and obtain community participation, which is essential for comprehensive, equity-centered research. While it was easier to assemble community partners in North Durham, it was considerably more challenging to form partnerships with groups and established organizations in South Durham, primarily due to mistrust regarding scientific data and the potential for resulting social and financial impacts. Authentic relationship building over time and engaging trusted intermediaries in the community can help mitigate this impact on the performance of research and the potential for bias in the work. One aspect of this trust-building process includes reporting the findings of this research through approaches that are appropriate for community members, and ongoing engagement on interventions and future work to support community education and self-determination.

In this study, we evaluated the levels and elemental speciation of PM from communities with increased asthma rates. Beyond the environmental-level characterization of PM levels and bulk elemental composition, health risk assessment can be advanced by gaining a deeper understanding of the contaminants contained in the particles and studying their exposure effects on model systems. Future studies will focus on the concentrations of specific contaminants such as microplastics. We will also use an in vitro respiratory cell model to study the toxicity of PM collected in Durham. Combined with the findings presented here, these data can better inform communities about the health risks posed by PM_2.5_ and by source emitters.

## Figures and Tables

**Table 1:** Comparison of health and demographic statistics between Census tracts within zip code 27701.

Area	CT 3.02	CT 14	NC
Asthma prevalence (percentile)	49	97	
Diabetes prevalence (percentile)	46	97	
Shortened life expectancy (percentile)	43	96	
Lowest median income (percentile)	61	98	
Social vulnerability index (SVI)	0.4717	0.9687	0.5
Disproportionality ratio (White)	1.09	0.07	1
Disproportionality ratio (Black)	0.50	3.68	1
Disproportionality ratio (Hispanic)	1.51	1.72	1
Disproportionality ratio (some other race)	1.42	2.36	1

## Data Availability

The original contributions presented in this study are included in the article. Further inquiries can be directed to the corresponding author.
